# Supplementary material for: Artificial transneurons emulate neuronal activity in different areas of brain cortex
Source: Nat Commun. 2025 Aug 7;16:7289. doi: 10.1038/s41467-025-62151-9 (PMC12332047; doi:10.1038/s41467-025-62151-9)
Supplement: Supplementary file 1 — Supplementary Information [file 41467_2025_62151_MOESM1_ESM.pdf]

Supplementary Information (SI) for the manuscript

Artificial transneurons emulate activity in different areas of brain cortex

Rivu Midya, Ambarish S. Pawar, Debi Pattnaik, Eric Mooshagian, Pavel Borisov, Thomas D. Albright, Lawrence H. Snyder, R. Stanley Williams, J. Joshua Yang#, Alexander G. Balanov#, Sergei Gepshtein#, Sergey E. Savel'ev#

1. Stochastic and deterministic dynamical equations for transneuron

The physical model of the transneuron is described using stochastic differential equations (see Methods), which have been demonstrated to replicate the findings of numerous prior experiments involving diffusive memristors [27–29, 61]. However, the interplay between stochastic and deterministic forces complicates the differentiation between self-sustained oscillations and noise-induced dynamics.

Here, we qualitatively justify the use of purely deterministic system dynamics to uncover the self-sustained mechanisms underlying spiking behaviour in artificial neurons. To achieve this, we consider a purely deterministic one-cluster model with no external noise, $D_V = 0$. In this case, stochasticity originates from thermal forces acting on the Ag-cluster only. The influence of thermal noise on the cluster's dynamics can be partially replicated by substituting stochastic forces with deterministic thermophoretic and/or thermoelectric forces, as discussed in references [30] and [31] in the main manuscript. Indeed, if we assume that the slowest component of the dynamics is related to the diffusion of the Ag cluster, we can substitute the static solutions of the equations (1b, 1c)

$$T(x) = T_0 + \frac{V^2}{C_{th}\kappa R(x)} = T_0 + \frac{V_{ext}^2 R(x)}{C_{th}\kappa (R(x)+R_{ext})^2} \quad \text{(S1)}$$

to Eq. (1a) and then study the Ag-cluster dynamics in the case of a position-dependent diffusion constant [59]. Therefore, the memristor internal temperature depends on the nanoparticle location, and thermal fluctuations can be replaced by an effective force, which depends on the temperature gradient. The temperature-gradient force plays a key role in the dynamics of the memristor. For example, as was numerically demonstrated in [59], this force produces a dynamical transition in the diffusive memristor: as the voltage increases, a nanocluster trapped in the energy minimum at low voltages jumps to the location with a minimum temperature, which can be attributed to forces arising from entropy gradients. To estimate this force, we assume that both V and T experience deviations from their mean trajectories $V_m(x(t) + \delta x)$ and $T(x(t) + \delta x)$ due to thermal noise. Introducing the spatial derivatives (gradients) of the memristor voltage $V' = [\frac{\partial V(x(t)+\delta x)}{\partial \delta x}]_{\delta x=0}$ and temperature $T' = [\frac{\partial T(x(t)+\delta x)}{\partial \delta x}]_{\delta x=0}$, we derive four coupled deterministic dynamical equations presented in the main manuscript (Eqs. 2a-d). The dimensional version of Eqs. 2a-d used in our simulations is derived in Section 2 of SI.

2. Normalisation of equations

To minimise the number of parameters and simplify model equations (1a-1c) and (2a-2d), we use the following normalisations:

$$t = \tau\frac{\tilde{t}}{30}, x = L\frac{\tilde{x}}{2}, R = R_0\,\tilde{r}(x_1, \dots), R_{ext} = R_0\tilde{R}_{ext}, \quad \text{(S2a)}$$

$$T = \left(\frac{15\eta L^2}{2\tau k_B}\right)\tilde{T}, V = \left(\frac{19\eta L^2}{2q\tau}\right)\tilde{V},\ T_0 = \left(\frac{15\eta L^2}{2\tau k_B}\right)\tilde{T}_0, \quad \text{(S2b)}$$

$$D_V/2 = T_V = \left(\frac{3\eta^2 L^4}{\tau q^2}\right)\tilde{T}_V, U = \left(\frac{15\eta L^2}{2\tau}\right)\tilde{U}, \quad \text{(S2c)}$$

$$\lambda = L\tilde{\lambda}/2,\ \tilde{\kappa} = \frac{\kappa\tau}{30}. \quad \text{(S2d)}$$

We set $\tilde{\lambda} = 0.12$ and the potential shown in Fig. S1A (the exact profile of the potential $\tilde{U}(\tilde{x})$ is provided in the shared data). In equations (1b) and (2b), $C_{th} = \frac{2.22\eta L^2 k_B}{q^2 R_0}$ resulting in $\tilde{c}_T = 0.18$ in Eq. (S3b) for all our simulations, except for the simulations in Figs. S2B and C, where we use $C_{th} = \frac{20\eta L^2 k_B}{q^2 R_0}$, resulting in $\tilde{c}_T = 0.02$. The integration step for the Euler–Maruyama method is 10^{-5}. Other parameters are given in the table below: for each figure in the main text and supplementary materials. With this normalisation, Eqs. (1a-c) reduce to:

$$\frac{d\tilde{x}_i}{d\tilde{t}} = -\frac{\partial \tilde{U}(\tilde{x}_i)}{\partial \tilde{x}_i} + 0.63(3)\tilde{V} + \sqrt{2\tilde{T}}\,\xi_i(\tilde{t}), \quad \text{(S3a)}$$

$$\frac{d\tilde{T}}{d\tilde{t}} = \tilde{c}_T \frac{\tilde{V}^2}{\tilde{r}(x_1, \dots x_N)} - \tilde{\kappa}\left(\tilde{T} - \tilde{T}_0\right), \quad \text{(S3b)}$$

$$30\frac{d\tilde{V}}{d\tilde{t}} = \tilde{V}_{ext} - \left(1 + \frac{\tilde{R}_{ext}}{\tilde{r}(\tilde{x}_1, \dots \tilde{x}_N)}\right)\tilde{V} + \sqrt{2\tilde{T}_V}\varsigma_V(\tilde{t}) \quad \text{(S3c)}$$

With

$$\tilde{r} = \cosh\left(\tilde{x}_1/0.12\right)$$

for one particle, and

$$\tilde{r}(\tilde{x}_1, \tilde{x}_2) = \left(e^{\tilde{x}_1/0.12} + e^{-\tilde{x}_2/0.12} + e^{(\tilde{x}_2 - \tilde{x}_1 - 1)/0.12}\right)/2$$

for two particles in the bottleneck (gap). For Fig. S1E, the charges of two particles were different, reflected by the different coefficients 0.57 and 0.95 for particles *i*=1 and *i*=2, instead of 0.63(3) in Eq. S3a. Comparison Fig. 4J and Fig. S1E reveals that variations in particle charges can further suppress spiking in the noise-induced regime. All the simulations begin with the particles attached to the pillar close to the potential minima $\tilde{x}_1 = -1 + 0.0001$, $\tilde{x}_2 = -1 + 0.0002$, $\tilde{T} = \tilde{T}_0$, $\tilde{V} = 0$.

By normalizing Eqs. (2a)-(2d), we obtain the following dimensionless ordinary-differential equations:

$$\frac{d\tilde{x}}{d\tilde{t}} = -\frac{\partial \tilde{U}}{\partial \tilde{x}} - 0.63(3)\tilde{V} - \tilde{q}_T \tilde{T}' \quad \text{(S4a)}$$

$$\frac{d\tilde{T}'}{d\tilde{t}} = \tilde{c}_T \frac{2\tilde{V}\tilde{V}'\tilde{r} - \tilde{V}^2\tilde{r}'}{\tilde{r}^2} - \tilde{\kappa}\tilde{T}' \quad \text{(S4b)}$$

$$30\frac{d\tilde{V}}{d\tilde{t}} = \tilde{V}_{ext} - \left(1 + \frac{\tilde{R}_{ext}}{\tilde{r}(\tilde{x})}\right)\tilde{V}, \quad \text{(S4c)}$$

$$30\frac{d\tilde{V}'}{d\tilde{t}} = -\left(1 + \frac{\tilde{R}_{ext}}{\tilde{r}(\tilde{x})}\right)\tilde{V}' + \frac{\tilde{R}_{ext}\tilde{r}'(\tilde{x})}{[\tilde{r}(\tilde{x})]^2}\tilde{V}. \quad \text{(S4d)}$$

Here we use an additional normalisation: $T' = (15\eta L/\tau k_B)\tilde{T}'$, $q_T = k_B \tilde{q}_T$, $V' = (19\eta L/q\tau)\tilde{V}'$, and the spatial derivative of dimensionless resistance $\tilde{r}$ was set to $\tilde{r}' = \sinh\left(\tilde{x}_1/0.12\right)/0.12$. Initial conditions were the same as in stochastic simulations and $\tilde{T}' = 0$, $\tilde{V}' = 0$ at $t = 0$.

Supplementary Table 1. Parameters of simulations for different figures; the colour of the values matches colour of the graph in the figure.

Supplementary Table 1	Fig.4 D/E/F	Fig.4 1G/H	Fig.4 2J	Fig.5A,B	Fig.5 C/D/E	Fig. 6BDH	Fig 7A	Fig. 7B/C/D	Fig. S1F	Fig. S1G
Clusters number	1	1	2	1	1	1	1	1	2	2
$\tilde{\kappa}$	0.9	0.9	0.9	0.9	0.9	0.9	0.9	0.9	0.9	0.9
$\tilde{R}_{ext}$	500	500	500	Varies: 50-5000	2500/50 /3200	500	500	500	1000	1000
$\tilde{T}_0$	0	0	0	0	0	0	0	0.003	0; 0.002	0.002; 0
$\tilde{T}_V$	0	0	0	0	0	0	0	0	0; 0.002	0; 0.002
$\tilde{V}$, $\tilde{V}_{th} = 18$	10/30 /195	As in Fig1 DEF	varies: 3.6-81	varies 0-158	24/106 /37	$\tilde{V}_{DC}$=10	0-250	$\tilde{V}_{DC}$ =20 $0.45 < \frac{\tilde{V}_{AC}}{\tilde{V}_{DC}} < 0.6$ (B) $\frac{\tilde{V}_{AC}}{\tilde{V}_{DC}}$ for C/D in E/F	varies 0-158	varies 0-158

Supplementary Table 1	Fig S1C, D($\tilde{q}_T = 1$)	Fig S1E $\tilde{q}_1 = 1.5\tilde{q}$ $\tilde{q}_2 = 0.9\tilde{q}$	Fig.S2 B/C	Fig. S4	Fig. S7	Fig. S8B
Clusters number	1	2	1	1	1	1
$\tilde{\kappa}$	0.9	0.9	0.1	0.9	0.9	0.9
$\tilde{R}_{ext}$	500	500	500	500	500	500
$\tilde{T}_0$	0	0	0.04/0.4	0, 0.007, 0.02, 0.04, 0.1, 0.2	0	0
$\tilde{T}_V$	0	0	0	0	0	0
$\tilde{V}$, $\tilde{V}_{th} = 18$	10/30 /195	varies: 0.2-2.15	158	10	$\tilde{V}_{DC} = 195$; $0.3 < \tilde{V}_{AC}/\tilde{V}_{DC}$<1	10 ($\tilde{V}_{AC}/\tilde{V}_{DC}$=0.1 and 0.3) 20 ($\tilde{V}_{AC}/\tilde{V}_{DC}$=0.5 and 0.7)

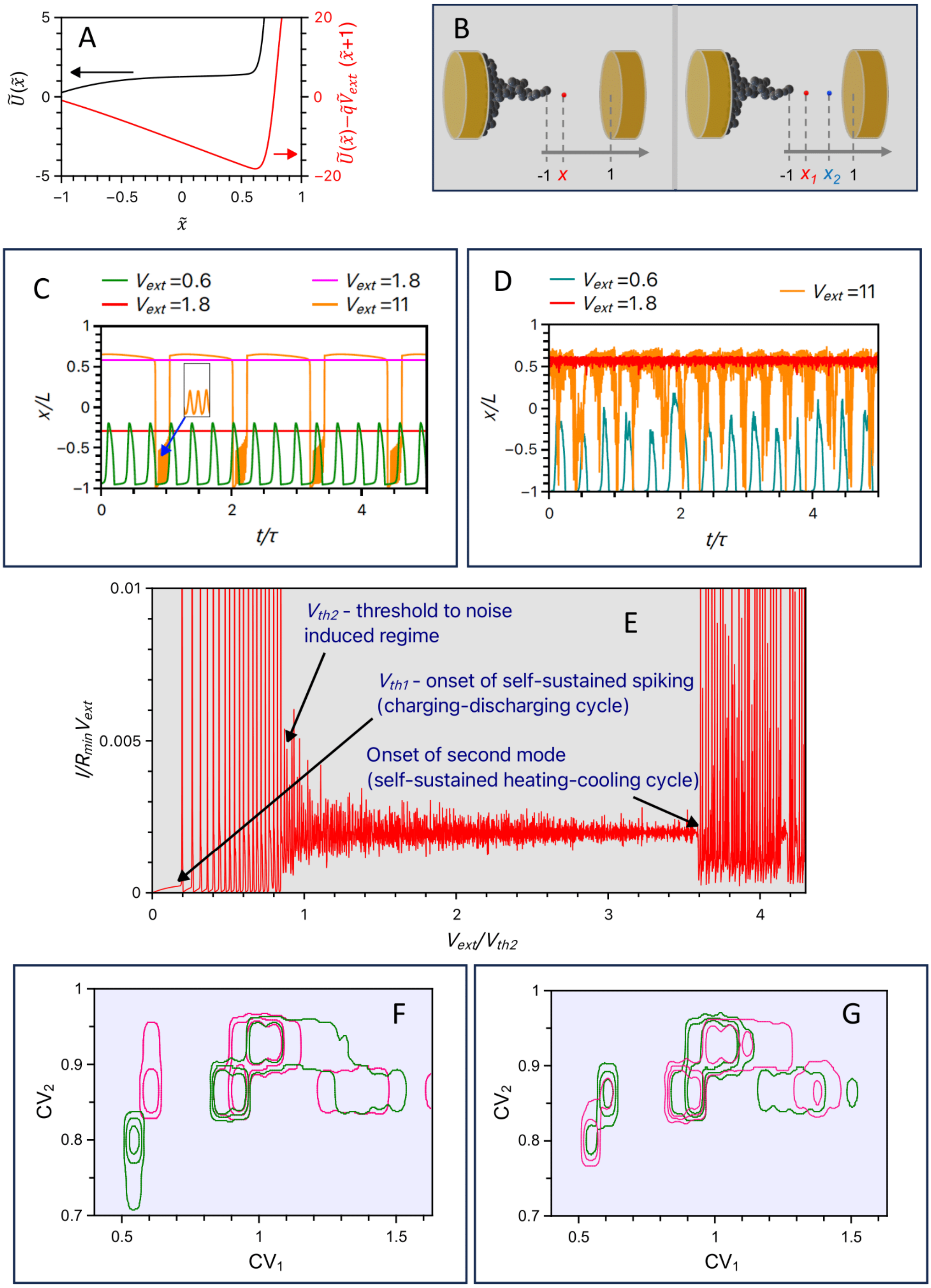

Fig. S1. (A) A voltage-biased potential energy $\tilde{U} - 0.63\tilde{x}\tilde{V}$ used to simulate stochastic dynamics of nanoparticles diffusing in the simulated memristor at zero voltage bias (black curve) and at high external voltages (red curve, $\tilde{V} = 4$). Without voltage bias, the particles are attracted by the tip of the filament (at $\tilde{x}$=-1), while at high voltages the particle diffuses to the minimum (at x=0.6), which is closer to the memristor terminal at $\tilde{x}$=1. This simple potential profile allows to reproduce all experimentally observed dynamical states of the artificial neuron with a diffusive memristor. (B left) A sketch of an Ag particle (red) at position $\tilde{x}$ drifting between the tip of the filament (dark circles) at $\tilde{x}$=-1 and the memristor terminal. (B right) is the same as (B left), but drawn for the case of two particles (one in red one in blue) diffusing in the gap. The yellow cylinders for both (B left) and (B right) represent Pt memristor terminals, one at the base of the filament and the other at $\tilde{x}$=1. Particle trajectory simulations obtained using deterministic (2a-d) and stochastic (1a-c) equations are illustrated in (C) and (D), respectively. Green, red/magenta, and orange curves correspond to, respectively, the regions I, II (with two fixed points), and III of Fig. 7A in the main manuscript. (E) The simulated spiking for slow varying voltage similar to Fig.4J, but for two particles with slightly different induced charges (see details of simulations discussed just after the set of Eqs. S3); (F, G) The distribution of (CV_1, CV_2) points is shown for a transneuron with different types and levels of noise (Supplementary Table 1, colours of curves match font colours in the table).

3. Self-sustained oscillations of artificial diffusive neurons

To identify the range of voltages where the artificial neuron exhibits self-sustained oscillations, we perform additional simulations using the one-particle dimensional stochastic model (1a-c) [normalised as Eqs. (S3a)-(S3c)] and the deterministic model (2a-d) [normalised as Eqs. (S4a)-(S4d)]. For all the numerical simulations, we use a phenomenological potential $\widetilde{U}$ *(*$\tilde{x}$*)* (black line in Fig. S1A; the data of the potential profile are shared in the data repository). This potential has one minimum at the tip of the nearly completed conducting filament (i.e., at $\tilde{x}$=-1), which represents attraction of Ag-particles to the filament (sketched in Fig. S1B). Indeed, when the particle is attached to the tip of the filament, the total surface area separating silver and $Si0_2$ is smaller than in the case when the particle is detached. This means that the surface energy proportional to this surface area has a minimum when the particle touches the pillar tip (Ref. [28] in the main manuscript). When a strong-enough voltage is applied, the total potential $\widetilde{U} - 0.63\tilde{x}\widetilde{V}$ is tilted (red line in Fig. S1A), pushing the particle away from the tip of the filament.

The spiking activity illustrated in Fig. S1C-D corresponds to the values of the applied constant voltages chosen in the regions I, II and III of the dynamical phase diagram displayed in Fig. 7A. For the voltage region I, the green curve in Fig. S1C represents the self-sustained oscillations obtained in the deterministic model, while the corresponding stochastic simulations (shown by the green curve in Fig. S1D) exhibit quite regular spiking slightly perturbed by noise. This ''charging-discharging'' cycle originates from the periodically charging capacitor, which causes the voltage drop across the memristor in its high-resistive state to exceed the threshold (the charging part of the cycle). As a result, the memristor switches to its low resistive state followed by a spike of current and a fast voltage decrease (the discharging part of the cycle) destroying the conductive filament and setting memristor to its high resistive state. Then the cycle repeats, starting from the charging part. A close biological analogue of this manner of spiking is the integrate-and-fire mechanism.

For the voltage region II, no spiking is observed in deterministic simulations, as the nanoparticle is attracted to the fixed points at either $\tilde{x} \approx 0.6$ (magenta curve) or $\tilde{x} \approx -0.35$ (red), depending on the initial conditions. Stochastic simulations show fluctuations of the nanoparticle near $\tilde{x} \approx 0.6$ (red curve) due to its larger basin of attraction, as compared to the point $\tilde{x} \approx -0.35$. Physiological studies demonstrate that only the stimulus within the neuron's receptive field can elicit spiking above the level of spontaneous activity. In this regard, the infrequent spiking in the noise-induced spiking mode can be interpreted as the spiking activity observed when the stimulus falls outside of the transneuron's receptive field.

At higher voltages (in region III), the self-sustained oscillations of the nanoparticle between positive ($\tilde{x} > 0.6$) and negative ($\tilde{x} \approx -1$) locations, with a relatively large period, were obtained by simulating the deterministic model (Eqs. S4a-d; the orange curve in Fig. S1C). When the particle is at $\tilde{x} > 0.6$, the system slowly heats up, similar to how resources accumulate in biological neurons before intensive bursting. As soon as the memristor is hot enough, the particle is pushed by thermal fluctuations to a low resistive state, the capacitor discharges rapidly, and the Ag-clusters move toward the edge of the bottleneck ($\tilde{x} \approx -1$). Then, intensive spiking begins. When the nanoparticle is in the region between $\tilde{x}$=-1 and 0, it executes a series of higher-frequency oscillations with a growing amplitude (inset in Fig. S1C) before it returns to the position $\tilde{x} \approx 0.6$, and a new heating cycle begins. These oscillations yield spike trains or bursting spiking behaviour. All of these features were also found in our stochastic simulations (e.g., see the orange curve in Fig. S1D) indicating that we still observe self-sustained oscillations in the presence of noise. In artificial neurons, heating-cooling cycles result in a modulation of the diffusion constant of Ag-clusters due to temperature variations (because, in our formalism, diffusion constant is proportional to Ag-cluster temperature). Although such high-temperature modulations are not observed in biological systems, variations in diffusion constants can significantly influence the dynamics of biological neurons, drawing an analogy to the heating-cooling cycles of

spiking observed in artificial neurons [Amir et al., Oscillatory mechanism in primary sensory neurones, Brain 125, 421 (2002), and Siegelbaum & Tsien, Modulation of gated ion channels as a mode of transmitter action, Trends in Neurosciences 6, 307 (1983)]. In biological neurons, variations in diffusivity may arise, for instance, due to changes in channel shape influenced by fluctuations in ATP or oxygen concentrations in the neuronal environment. These concentrations are, in turn, affected by the spiking activity of the neurons. Specifically, ATP and oxygen levels decrease as they are consumed during neuronal activity, leading to a subsequent slowdown in spiking. Once spiking activity diminishes, ATP and oxygen levels recover, allowing the cycle to repeat.

4. Internal (diffusive) and external (network) noises and their influence on system dynamics

Detailed comparison of how external and internal noises influence the pseudo-deterministic system dynamics requires a separate study of a more complicated neuromorphic circuits with multiple transneurons. Here, we only consider how these noises influence the stochastic characteristics of activity: CV_1 and CV_2. Figures S1F-G present contour-plots illustrating local maxima in the distribution of (CV_1, CV_2). The figures indicate that additional noise sources (either arising from voltage fluctuations or attributed to additional bath temperature T_0) mostly affect the quite regular spiking with CV_1 and CV_2, whose values are comparable to those of PRR biological neurons.

First, we simulated transneuron spiking with $T_0 = D_V = 0$ and $R_{ext}/R_0 = 1000$ for different applied voltages V_{ext}. The corresponding distribution of (CV_1, CV_2) values is shown in Fig. S1F by the olive contour-plot. The two distinctive maxima in the (CV_1, CV_2) distributions correspond to the more regular PRR-like spiking [for (CV_1, CV_2) around (0.55,0.6)] and the less regular (more stochastic) MT-like and PM-like spiking (for CV_1 spanning from 0.9 to 1.5).

Next, a transneuron spiking with (CV_1, CV_2) presented in Fig. S1F with the magenta contour-plot was simulated with both additional internal and external noises originated from the thermal bath and neural network (Supplementary Table 1). The cloud of (CV_1, CV_2) points in the distribution corresponding to MT-like spiking is slightly shifted away (and now is detached) from the points corresponding to PM-like spiking, even as both regimes still occupy adjacent areas in the (CV_1, CV_2) space. The cloud of (CV_1, CV_2) points in the distribution that corresponds to PRR-like spiking shifts more noticeably to higher values of CV_1 and CV_2. However, shifting the cloud corresponding to PRR-like simulated activity away from the (CV_1, CV_2) area that corresponds to biological PRR neurons requires a much stronger noise than used in the simulation. This observation highlights the robustness of all the (CV_1, CV_2) transneuron clouds associated with the stochastic behaviours of biological neurons in PRR, MT, and PM.

We have also compared the specific influence of the internal thermal bath alone, and of the external voltage noises alone, on the distribution of (CV_1, CV_2) points for transneuron spiking (Fig. S1G, olive and magenta curves, respectively). The simulations show that adding either of these noise sources alone affects the shape of the (CV_1, CV_2) distribution in a way similar to the case where both sources are present (compare Fig. S1F and Fig. S1G). Namely, the PRR-like cloud of (CV_1, CV_2) points shifts to higher values CV_1 and CV_2, and the MT-like and PRR-like clouds of (CV_1, CV_2) are divided into several islands. This is evidence that main findings of this study are robust to different types of noise and different noise intensities.

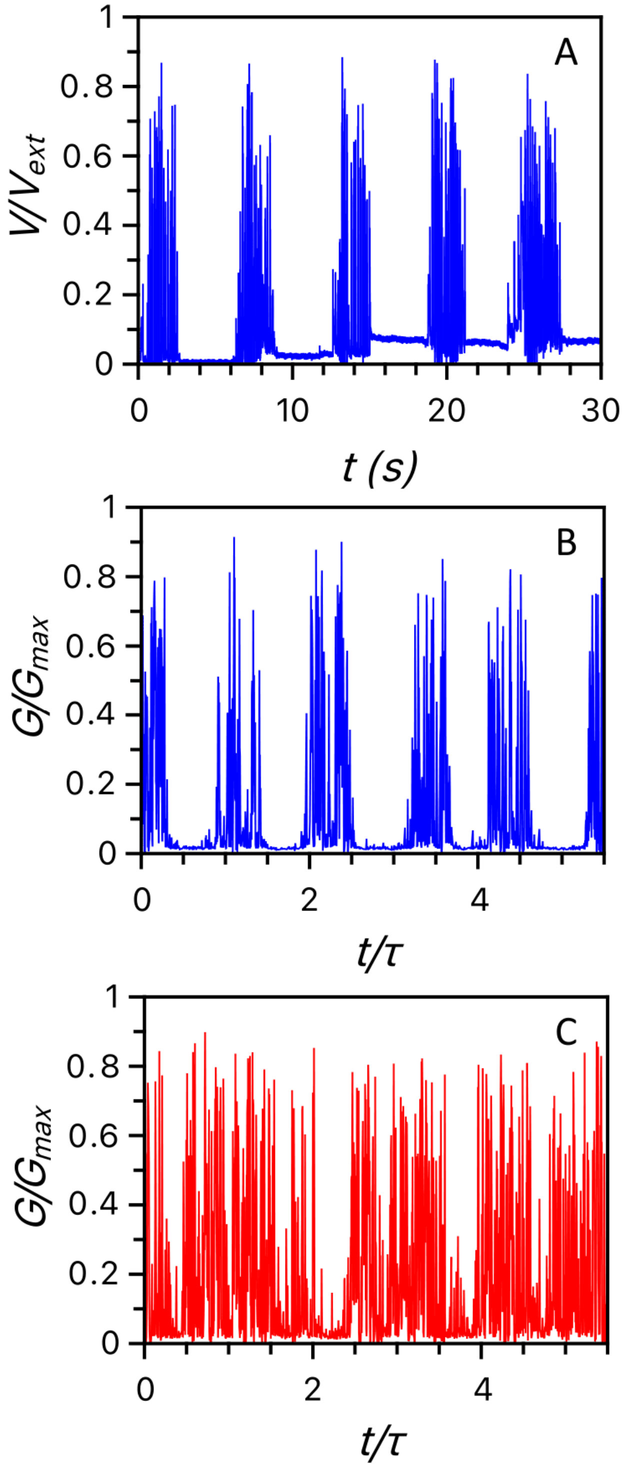

Fig. S2 (A) Experimentally measured bursting activity, which is voltage across the memristor normalised by the applied voltage. (B) Simulated bursting for the same parameters as in Fig. S1D (orange curve) but with κ and $\tilde{c}_T$being nine times lower than in Fig. S1D. (C) The same as panel B yet at higher temperatures (see Supplementary Table 1). Blue and red traces represent, respectively, the situations with low and high noise in the system.

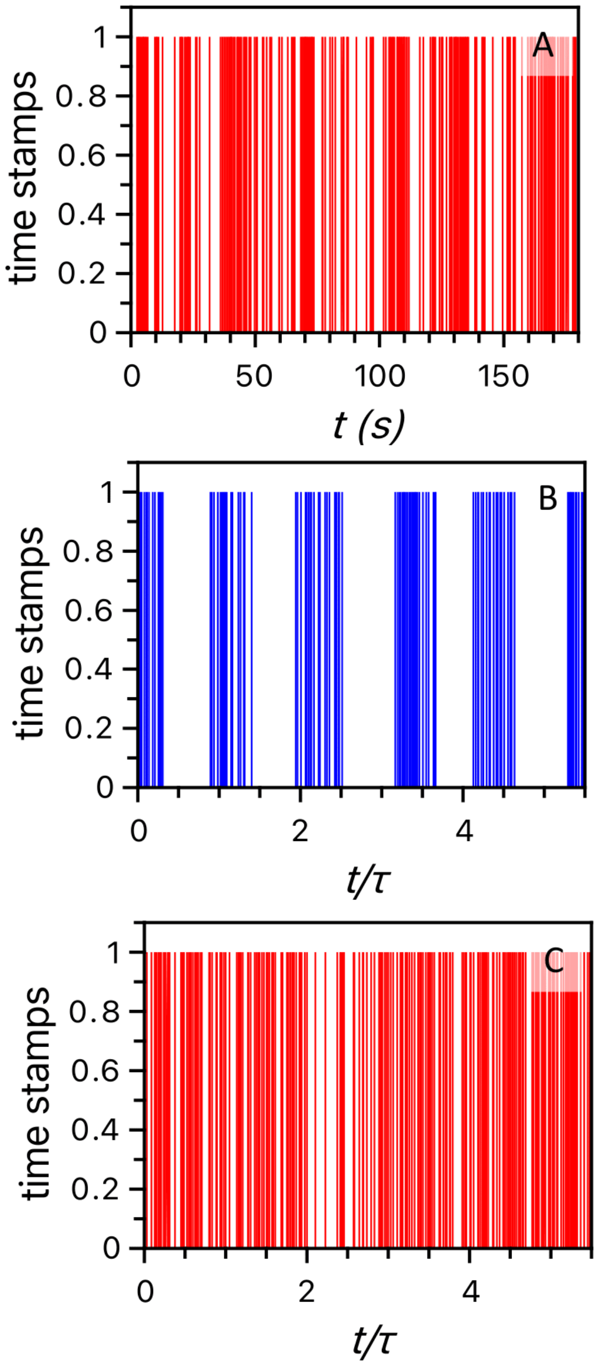

Fig. S3 (A) Timestamps of spikes in a bursting biological neuron (sampled from the data shown in Fig. 5 of the main text). (B-C) Timestamp of spikes obtained using the threshold of G/G_{max}=0.1, corresponding to the simulated data in Figs. S2B and S2C, respectively.

5. Bursting of transneurons and biological neurons: measurements and simulations.

Depending on the range of voltage V_{ext}, the cooling constant κ, and the load resistance R_{ext}, the observed and simulated activity at higher voltages has the form of either the partly overlapping spiking trains (as in Fig. 4C, F) or separate bursts of spiking (Fig. S2A-B). The lower the value of the cooling constant κ, the longer is the time interval between the successive spiking trains. These separated bursts are expected when the duration of heating needed to raise the temperature of Ag-clusters is much longer than the mean inter-spike interval. In this case, the memristor will stay in the intermediate resistive state for a long time (i.e., in the state where the cluster is stuck near the point of $\tilde{x} \approx 0.6$) with no spiking, resulting in the quiet intervals between bursts. The temperature will slowly rise, reaching the energy of the barrier that separates spiking and non-spiking dynamics. As soon as the temperature is high enough, thermal fluctuations push the memristor to its high resistive state in which the Ag-cluster is attached to the tip of the filament. In this state, the Ag-cluster starts to move repetitively between the tip and the middle of the gap, switching the memristor between the high and low resistive states, producing charging-discharging oscillations of the memristor's capacitor, and generating current spiking within a single train. In agreement with this analysis, the well separated spiking trains (Fig. S2B) have been observed in our simulation when the value of κ is lower than that in Fig. 4C. The experimentally observed bursting portrayed in Fig. S2A demonstrates similar spiking trains with long quiet intervals between them. (The evolution of bursting with the applied voltage is shown in Fig. 3.)

We also represent spiking by means of timestamps (Fig. S3B-C), with the spikes registered when the simulated signal, G(t), exceeds the threshold of G/G_{max}=0.1 and $G_{max}=1/R_0$. According to our simulations, an increase of the bath temperature T_0 results in noisier bursting (Fig. S2C), which corresponds to the less regular spiking bursts in the timestamp picture (Fig. S3C). In the latter figure, the vertical lines of equal height are shown at the instants of spike occurrence. The simulated noisy bursting (Fig. S3C) has common features with the bursting of premotor neurons illustrated in Fig. S3A. This suggests that tuning the bath temperature by additional heating or cooling the chipset with memristive device allows diffusive transneurons to emulate spiking of biological neurons at different levels of stochasticity.

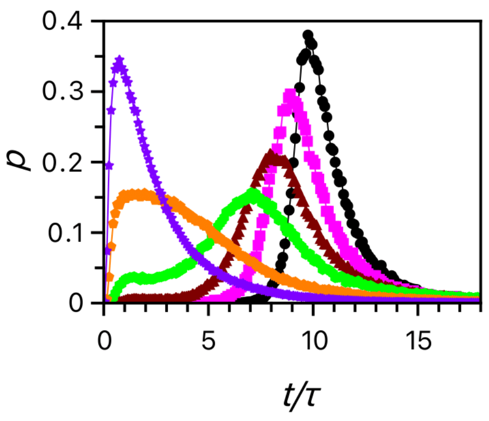

Fig. S4 Simulated evolution of the distributions of ISIs in an artificial neuron for parameters corresponding to Fig. S1D (green curve) with the temperature of the bath T_0 increasing (see Supplementary Table 1in which font colours correspond to the data point colours used in this figure). At low temperatures (black, magenta, and brown symbols) ISI probability densities have a clear peak corresponding to a self-sustained spiking mode (as in Fig. 4A, D). The distributions are well approximated by Gamma distributions. At higher temperatures (orange and violet symbols), the distribution evolves towards a Poisson-like distribution with the most probable value of ISI approaching zero.

6. Control of stochasticity by bath temperature of artificial diffusive neurons

As seen in the previous section (Section 5 of SI) of Supplementary Information, bath temperature significantly affects the noisy pattern of bursting (Fig. S2, S3) in the cooling-heating self-oscillating regime of the system (regime III in Fig. 7A and orange curves in Fig. S1C-D). Here we demonstrate how temperature affects ISI distribution for the first oscillating mode (PRR-like quite regular spiking) related to charging-discharging the capacitor in the regime I in Fig. 7A (illustrated by the spiking pattern in Fig. 4A, D and by the green curve in Fig. S1D).

Most simulations presented in the main text were performed for the background temperature much lower than the temperature change due to Joule heating (i.e., we neglected T_0). The reason is that the voltage drop occurs across a very small gap between

the filament and the terminal (see Fig. S1B), resulting in a high local temperature of the Ag-cluster in the gap, as compared to the bath temperature. Here we consider how increasing the background temperature affects the statistics of inter-spike intervals (Fig. S4). At low temperatures, the ISI distribution has a pronounced maximum far from zero; here the ISI statistics are described well by the Gamma distribution, similar to the commonly observed ISI statistics in biological neurons (Ref. [36] in the main text). With increase of T_0, the noise in the system becomes more intense, which makes the self-sustained spikes more frequent and less regular. Therefore, as T_0 increases, the maximum of the distribution gradually shifts towards low ISI values and the distribution peak broadens. Simultaneously, an additional broad local maximum emerges at near-zero ISI, corresponding to the additional noise-induced spikes triggered by the large-enough fluctuations. For even higher values of T_0, the maximum corresponding to self-sustained oscillations is suppressed completely, and the distribution approaches the Poisson distribution. Thus, varying the background temperature allows for additional tuning of stochasticity in artificial diffusive neurons helping to emulate the desired statistical features of different types of biological neurons.

7. Dependence of stochastic characteristics on stimulus intensity or contrast

Spiking of biological neurons could be triggered ("evoked") by stimulation or it can be spontaneous. In our physiological experiments with visually stimulated MT neurons, the measured spiking is necessarily characterised by both spontaneous and evoked components. For more intensive

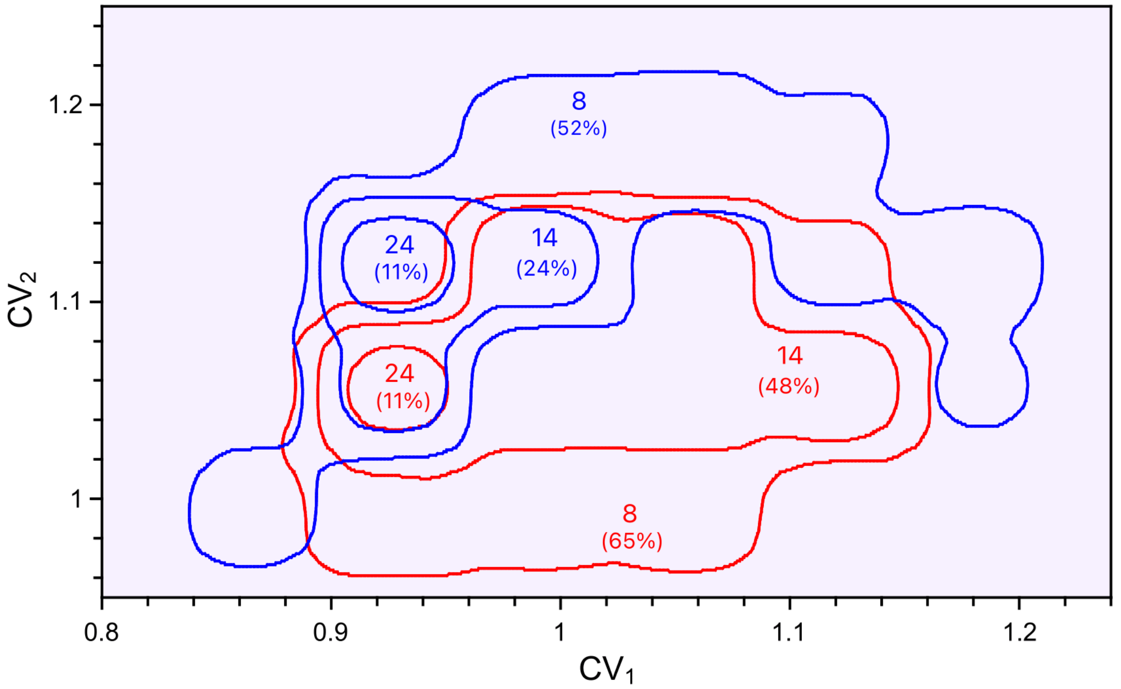

Fig. S5. Contour plots of the distributions $\rho(CV_1, CV_2)$ of (CV_1, CV_2) points for MT biological neurons under two combined contrasts of visual stimulation: the contour plot in blue for contrasts 0.5% and 1.9%, and the contour plot in red for contrasts 26.6% and 100%. The numerical values displayed near each closed contour correspond to the values of the ρ taken at the corresponding contours, while the per-cent values represent the probability that a (CV_1, CV_2) point is found inside the contour.

stimulation, the evoked activity increases, suggesting a method for estimating the relative influence of evoked and spontaneous spiking. Such analysis can be useful for comparing the activities of biological neurons and artificial transneurons. For the latter, the AC-voltage component can be attributed to stimulation, and the DC-voltage component can be seen as a driver of both spontaneous and evoked spiking, depending on the information coding protocols (e.g., Fig. 6 in the main text).

To understand how stimulus intensity influences spiking of biological neurons, we focus on how the activity of MT-neurons depends on visual stimulus contrast. Fig. S5 illustrates the (CV_1, CV_2) distributions for low- and high-contrast stimuli (blue and red contour-plots, respectively). To improve

statistical power of our analysis, we combine (CV_1, CV_2) distributions for two lowest contrasts (0.5% and 1.9%) and two highest contrasts (26.6% and 100%) of visual stimuli. It is evident that the contour-plots for high and low contrasts significantly overlap, and that the maximum of the distribution is shifted to lower values of CV_2 for higher- contrast stimuli, even as the maxima of both distributions are found at nearly the same values of CV_1. This indicates that, in MT neurons, the higher intensity of visual stimulation makes the values of the nearest interspike intervals slightly more similar to one another (increasing the local coherence of spike sequences) while having little effect on the overall ISI distribution. The significant overlap of the distributions demonstrates that the values of (CV_1, CV_2) are controlled mainly by the internal dynamics of different types of neurons, justifying our approach to accumulate all (CV_1, CV_2) values for each cortical area regardless of stimulus intensity.

8. Overlapping estimates of measured stochastic characteristics in transneurons and biological neurons.

By changing DC voltage and/or external resistance, the stochastic characteristic of a transneuron can be tuned to different areas in the (CV_1, CV_2) plane. To understand what fraction of biological neurons in cortical areas MT, PRR, and PM can be mimicked by transneurons, we first identify the convex hull (red shadowed region) of the measured points (shown in Fig. S6A as black circuits) for transneurons at different DC voltages and different external resistances and capacitances. We then count the number of (CV_1, CV_2) points for each type of biological neuron (MT, PRR, or PM) inside this region and divide the result by the total number of (CV_1, CV_2) points for the corresponding type of neuron. The resulting fractions of the overlap of MT, PRR, and PM (CV_1, CV_2) covered by the convex hull of the measured transneuron (CV_1, CV_2) points are 100%, about 70%, and 100% (see Fig. S6).

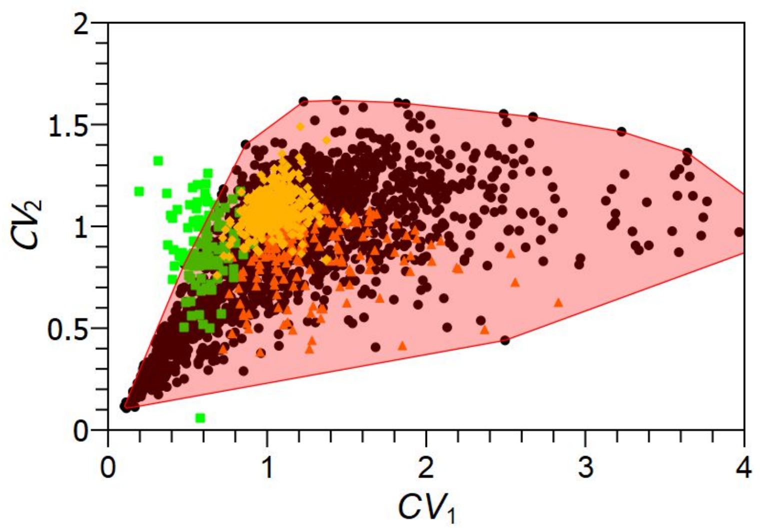

Fig. S6. The overlap of (CV1, CV2) points for the spiking of biological neurons across different cortical areas and transneurons measured at various external voltages, resistances, and capacitances. The red-shadowed convex hull plotted for the measured (CV1, CV2) data set for transneurons, encompasses 100% of the MT (CV1, CV2) points (yellow), approximately 70% of the PRR (CV1, CV2) points (green), and 100% of the PM (CV1, CV2) points (orange).

9. Selectivity of mode 2 spiking (cooling-heating oscillations)

Figs. 6C-D, G in the main text illustrate transneuron's selectivity in the quite regular spiking mode (such as in Fig. 4D). Selectivity manifests itself in a sharpening of the ISI distributions (Fig. 6C-D) when AC-

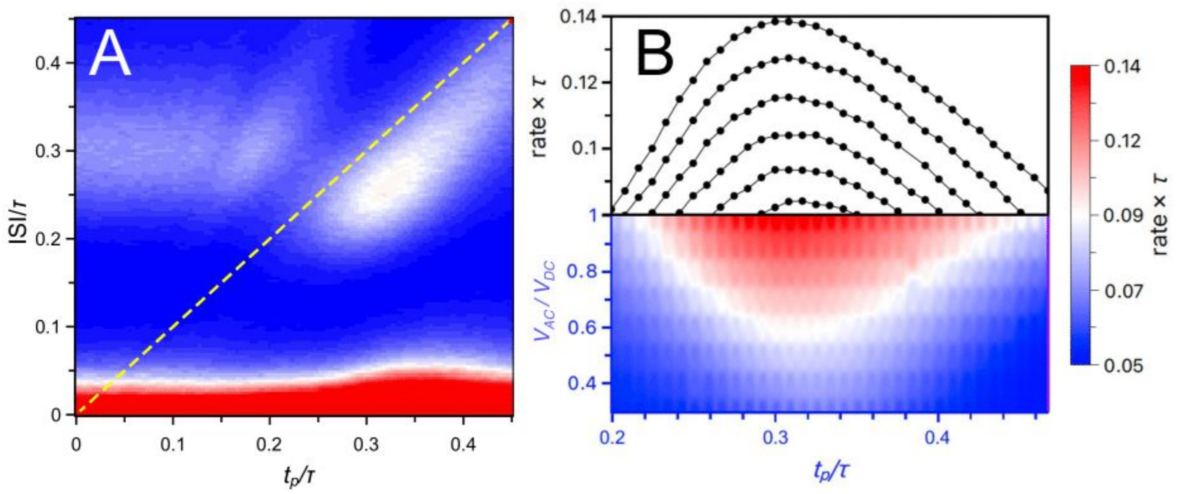

Fig. S7 (A) The simulated distribution of ISI intervals for the same parameters as in Fig. 6 but with the DC voltage corresponding to Fig. 4F. (B) The average spiking rate as a function of AC-period for the same parameters as in (A) and AC amplitude changing from 0.3 V_{DC} to V_{DC}. Simulation parameters are displayed in Supplementary Table 1.

voltage is applied with a period approaching to that of the neuron's mean ISI (which, in turn, is close to the natural spiking time [6]). Selectivity also manifests itself as the maximum spiking rate near the natural spiking time (Fig 6G). Such selectivity can be found also in other spiking regimes of the transneuron.

To demonstrate how neuronal selectivity changes with increasing stochasticity, in Fig. S7 we plot ISI distributions and the spiking rate for a more stochastic spiking regime (as in Fig. 4F, corresponding to stochastic noisy bursting). The ISI distribution shows that a large fraction of spiking occurs at short values of ISI (red region in Fig. S7A). Nevertheless, a less pronounced maximum of the distribution (as compared to Fig. 6D) is observed at larger values of ISI (white region) and is characterised by the intrinsic time scale. However, when the AC-period (represented by the yellow dashed line) reaches the intrinsic ISI time, the white cloud becomes more pronounces and it is "dragged" by stimulation along the yellow line. These features are similar to the case of selectivity reported in the main text (Fig. 6D). Moreover, when the AC-period is close to the natural (or most probable) ISI, the spiking at low values of ISI becomes more intense (indicated in Fig. S7A by the red "bump" in the distribution, at low values of ISI, at t_p of about 0.35τ). These effects cause the spiking rate to have a maximum (Fig. S7B), in close analogy to the behaviour of selectivity observed in Fig. 6G in the main text. Finally, with increasing stimulus intensity (the amplitude of AC voltage), we observe a shift of the period at which the spiking rate reaches its maximum, toward a lower value of the period (i.e., a higher AC-frequency). This observation is consistent with the nonlinear ''resonance'' drift of the maximum of spiking reported in the main text for the case of quite regular spiking (Fig. 6G) and for biological MT neurons (Fig. 6E).

10. "Beyond rate" information coding

Here, we focus on (a) how the self-sustained and noise-induced spiking regimes can be used for encoding and processing information "beyond spike rate" coding and (b) how stimulus contrast can influence spiking of transneurons. The common rate coding hypothesis holds that information about sensory stimuli is encoded in the firing rates of cortical neurons, while other statistical properties of

ISI sequences are not essential. This view of neural function competes with other information coding paradigms, such as temporal coding [Gerstner, W., Kistler, W. Spiking neuron models: single neurons, populations, plasticity. *Cambridge University Press* (2002)], in which spiking statistics other than rate play an important role.

In the present study of biological and artificial neurons, we observed that ISI statistics inconsistent with the simple notion of scaling of ISI distributions with the firing rate (Fig. S8A-C). Such a scaling assumes that the shape of the ISI distribution does not change significantly as stimulation increases [Reich, D., Victor, J., Knight, B. The power ratio and the interval map: Spiking models and extracellular recordings. *J. Neurosci.* **18**, 10090 (1998)]. However, if these distributions are significantly affected by stimulation, then ISI statistics can be used for encoding and processing of information in biological and artificial neurons. Our physiological measurements show that an increase of stimulus strength (e.g., an increase of stimulus luminance contrast) can significantly affect the shape of ISI probability distributions (Fig. S8A). As stimulus contrast increases, ISI distributions for most MT neurons become narrower with more pronounced peaks (as in Fig. S8A, right panel), implying a change of response toward a more regular spiking activity. Still, some neurons counterintuitively exhibit a shift toward a more stochastic behaviour, resulting in a lowering and broadening of ISI distributions (Fig. S8A, left panel).

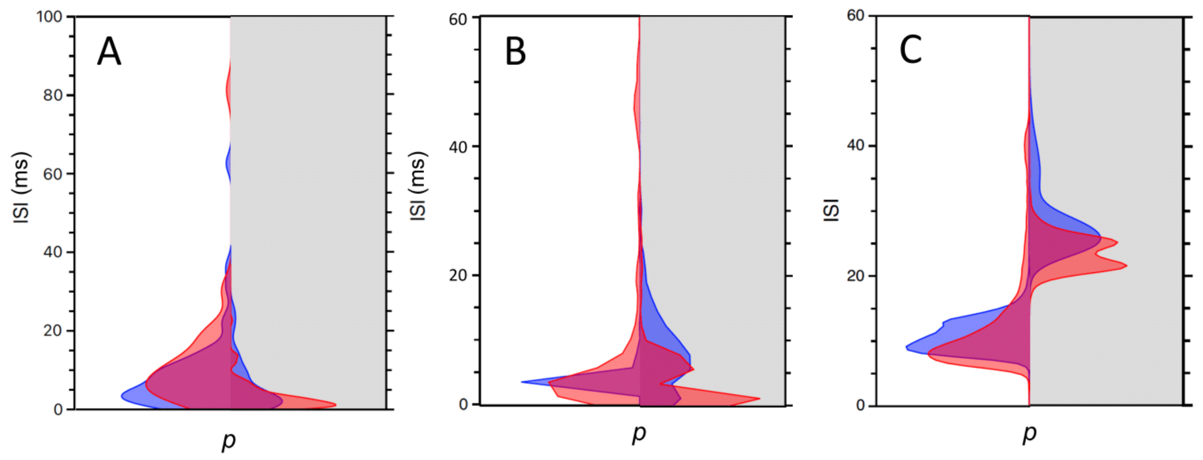

Fig. S8: Violin plots for high AC-amplitudes and contrasts are shown in red, and for low AC-amplitudes and contrasts are shown in blue, for two biological middle temporal (MT) neurons (A) that responded differently to an increase of stimulus strength, for the measured artificial neuron (B), and for the simulated transneuron (C). The DC voltages for measured and simulated artificial neurons were selected from the noise-induced spiking mode (as in Fig. 4B, E) and from the self-sustained spiking mode (as in Fig. 4A, D) shown, respectively, in the right and left panels of each violin plot.

Remarkably, we observe similar behaviour in our experiments with the artificial diffusive neuron driven by DC and AC voltage: $V_{ext} = V_{DC} + V_{AC} \cos \omega t$. To better understand the transformation of ISI distributions in artificial transneurons, we consider the cases where the transneurons are tuned to the boundary between noise-induced (Fig. 4B, E) and self-sustained (Fig. 4A, D) spiking regimes. If a transneuron is in the noise-induced mode at $V_{AC} = 0$, the arrival of a stimulus, $V_{AC} > 0$, can occasionally drive the neuron into the regime of self-sustained oscillation. Stronger stimuli (e.g., at higher AC voltage amplitude) increase the contribution of self-sustained oscillations in the neuron's activity, leading to a more regular spiking (see right panels of Fig. S8B for measurements, and Fig. S8C for simulation). However, if the neuron already dwells in the regime of self-sustained spiking at $V_{AC} = 0$, then increasing stimulus intensity eventually drives the neuron into the noise-induced regime, reducing regularity of spiking (see left panels of Fig. S8B for measurements, and Fig. S8C for simulation).

This method of controlling the ISI distribution transcends rate scaling, and it relies on the ability of the transneuron to change its spiking mode while excited by stimulation.

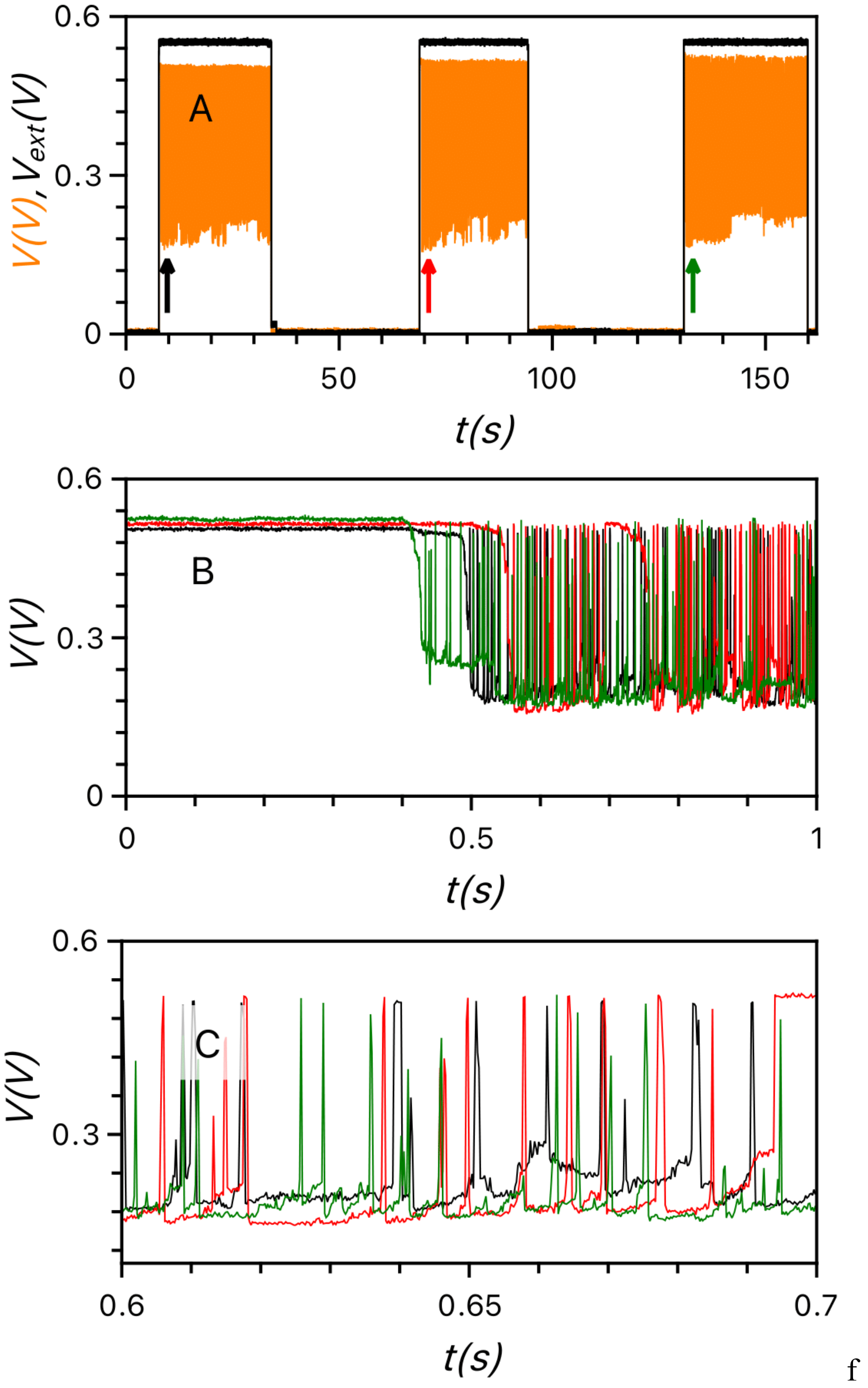

Fig. S 9. (a) Three pulses of external voltage (black line) of 30 sec separated by 35 sec of idle (zero applied external voltage) intervals and the corresponding spiking of voltage across the memristor (magenta line) for the transneuron with R_{ext}=65k Ohm and C= 50nF. Arrows indicate parts of spiking sequence enlarged in (b-c) with the colours of arrows corresponding to the colours of spiking curves in the panels below. This repetitive measurement shows that the transneuron's response is truly stochastic, with random delays of spiking after the beginning of each pulse (the time count in b-c starts from the moment of application of the corresponding voltage pulse) and irregular spiking for all three pulses with the not-coinciding peaks.

11. Repetitive measurements of transneuron spiking

In Fig. S9 we demonstrate a truly stochastic nature of spiking in transneurons. Namely, we measure system response to three voltage pulses separated by idle periods (when no external voltage is applied), which allow the system to relax and reset. Despite the same voltage applied in each pulse,

the spiking response to each pulse is characterized by different delays [28] relative to the pulse onset. The spiking patterns triggered by the pulses do not reproduce themselves even though they are characterized by similar stochastic features.

12. Small functional neuromorphic circuits with transneurons

Simple neuromorphic circuits capable to perform multi-signal comparison can be fabricated using modern thin-film equipment instead of wiring connections between artificial neurons (Fig. S10). This approach may resolve the problem of scalability of neuromorphic devices.

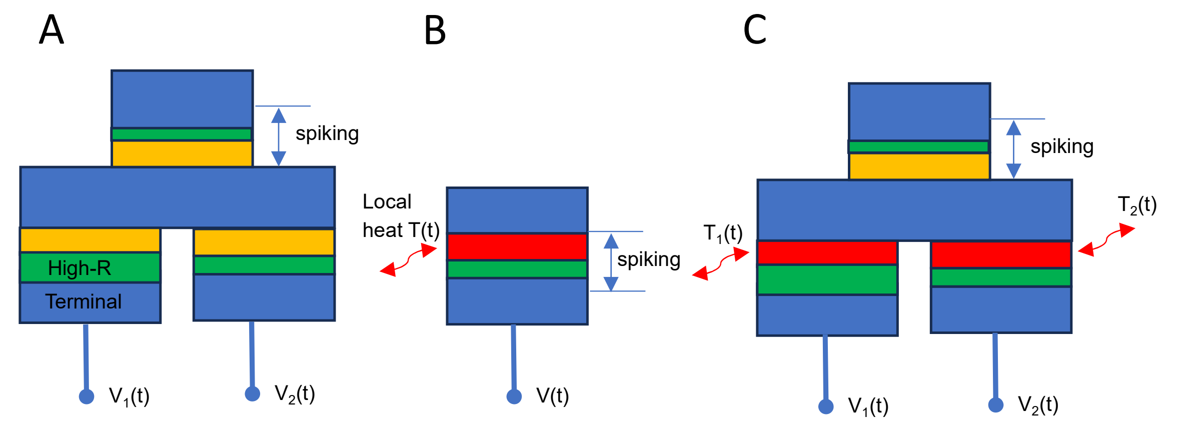

Fig. S10: (A) Three spiking artificial neuron block, which can be fabricated as a unit, rather than three separate neurons wired in a neuromorphic circuit. Blue layers correspond to memristors' terminals (e.g., Pt layers), green layers correspond to high-resistive materials used as external resistances, while yellow layers represent memristive layers. This block can be used for comparing two signals encoded in time-dependent voltages V_1 and V_2. This system can be used, for example, to compute the direction of sound or to evaluate the distance to an object. (B) One transneuron block with the red layer marking Ag-SiO diffusive memristor. This transneuron can perform two-signal comparison as in (A) encoded now in voltage and temperature signals as described in the main text. (C) Circuit arrangement for signal comparison performed by two transneurons with one artificial neuron used as readout. The signals (e.g., two for visual information and two for sound information) in T_1(t), T_2(t), V_1(t), V_2(t), can be used, e.g., for directional and distance estimation, thus identifying the location of an object.

Comparing the proposed transneurons with less tuneable artificial neurons based on volatile memristors (such as NbO devices), one can conclude that similar neuromorphic transneuron circuits could perform significantly more complex computations. Indeed, as shown above, one transneuron can perform two-signal comparison (Fig. 10SB), which requires a network of three artificial neurons (Fig. 10A). Such neuromorphic circuits can be used to estimate the distance from, or the direction to, the source of a signal, e.g., visual *or* auditory. A network of three transneurons (Fig. S10C) can potentially handle multimodal signals (e.g., visual *and* auditory), and thus greatly expand the capabilities of neuromorphic hardware.

The fabrication procedure for such devices could include the following steps. One could start with a clean and polished semiconducting substrate, such as a 500 µm GaAs wafer. On the polished and epitaxial side of the wafer, a gold sink layer (~200nm) could be deposited using sputtering or e-beam evaporation, to ensure smoothness and uniformity. Next, photolithography and liftoff could be performed to create two windows. The memristor layer can then be sputter deposited (e.g., Ag:SiOx, of approximately 50 nm), followed by deposition of a high resistive layer (e.g., Nichrome, of approximately 20nm), and then by deposition of a top gold contact layer (approximately 100 nm). After stripping the unexposed photoresist, two memristor mesa structures should be revealed on the polished side of the substrate with gold top electrodes for electrical connections. The substrate could then be bonded to a glass disk with the memristor side attached to the glass using wax. The backside of the substrate would then be thinned by lapping and polishing to a minimum thickness of approximately 50 µm. The next step would be to remove the entire GaAs substrate. A controlled etching of the GaAs substrate (e.g., bubble etch) can be performed until the gold layer becomes visible,

stopped once the entire gold sink layer is exposed. Further photolithography and deposition processes can be conducted to fabricate the third memristor layer on the backside of the now-etched wafer (on the gold sink layer). Finally, the fabricated device can be demounted from the glass disk, resulting in a flexible gold film with three memristor layers (as depicted in Fig. S10).

13. Details of physiological studies

Recordings in cortical area MT

Animals. Two adult male rhesus monkeys (*Macaca mulatta*) of ages 11 and 12 were used in this study. Experimental protocols were approved by the Salk Institute Animal Care and Use Committee and conform to US Department of Agriculture regulations and to the National Institutes of Health guidelines for the humane care and use of laboratory animals. Procedures for surgery and wound maintenance have been described in detail elsewhere [Dobkins, K.R., Albright, T.D., What happens if it changes colour when it moves?: the nature of chromatic input to macaque visual area MT. *J. Neurosci.* **14**, 4854 (1994) and Refs. [32, 34] in the main manuscript].

Apparatus. All visual stimuli were generated using Matlab (The MathWorks Inc, Natick) software using a high-resolution graphics display controller (Quadro Pro Graphics card, 1024x768 pixels, 8 bits/pixel) operating in a Pentium class computer. Stimuli were displayed on a 21-inch monitor (75 Hz, non-interlaced, 1024x768 pixels; model GDM-2000TC; Sony, Tokyo, Japan). The output of the video monitor was measured with a PR650 photometer (Photo-Research, Chatsworth, CA), and the voltage/luminance relationship was linearized independently for each of the three guns in the cathode ray tube.

Behavioural procedure. Monkeys were seated in a standard primate chair (Crist Instruments, Germantown, MD) with the head post rigidly supported by the chair frame. The task was to fixate a small (0.2 deg diameter) target in the presence of moving visual stimuli for the duration of each trial (500-2000 msec). The target was presented on a video display at a viewing distance of 57 cm in a dark room (<0.5 cd/m^2). The mean background luminance of the monitor was 15 cd/m^2. Eye position was sampled at 120 Hz using an infrared video-based system (IScan, Burlington, MA). The eye position data were monitored and recorded with the CORTEX program (Laboratory of Neuropsychology, National Institute of Mental Health, Bethesda, MD), which was also used to implement the behavioural paradigm and to control stimulus presentation. After eye position was maintained within a 2 deg window centred on the fixation target throughout the trial, animals were given a small (0.15 cc) juice reward.

Electrophysiological procedure. A craniotomy was performed to allow for electrode passage into area MT. Activity of single units was recorded in area MT using tungsten microelectrodes (3-5MΩ; Frederick Haer Company, Bowdoinham, ME), which were driven into cortex using a hydraulic micropositioner (model 650; David Kopf Instruments, Tujunga, CA). Neurophysiological signals were filtered, sorted, and stored using the Plexon (Dallas, TX) system. Visual responses were recorded from 139 directionally selective MT neurons in two awake, fixating macaque monkeys (74 and 65 neurons in Monkeys 1 and 2, respectively). We measured firing rates to stimuli at five to seven different levels of luminance contrast (0.05-100%) at the preferred spatiotemporal frequencies: five spatial frequencies (SF) and one to five temporal frequencies (TF). The different stimulus conditions and contrasts were interleaved in random order across trials.

Data resampling. For each neuron, the firing rates estimated in separate trials within each condition of stimulus frequency and contrast were resampled with replacement. The number of samples was ten (which is the number of trials employed in the experiments). Response functions were fitted to the resampled data using non-parametric polynomial regression, repeated for 500 iterations of resampling to estimate errors of peak SF within each condition. The errors were used to measure

differences between peaks across stimulus contrasts. A similar procedure was used to estimate errors of peak TF for each condition.

Recordings in cortical area PRR

Animals. All procedures conformed to the Guide for the Care and Use of Laboratory Animals and were approved by the Washington University Institutional Animal Care and Use Committee. Two male rhesus macaques (*Macaca mulatta*; Monkey 1 and Monkey 2) participated in the study.

Apparatus. Head-fixed animals sat in a custom-designed monkey chair (Crist Instruments, Hagerstown, MD) with a fully open front to allow unimpaired reaching movements. Visual stimuli were back-projected by an LCD projector onto a translucent Plexiglas screen mounted vertically, 40 cm in front of the animal. Eye position was monitored using the 120-Hz ISCAN eye-tracking laboratory (ETL-400). Touches were monitored every 2 ms using capacitive sensors, mounted at the home pads and behind the Plexiglas projection screen. Touch positions on the screen were organized in a 3x3 grid centred on the fixation point. Plexiglas dividers were mounted on the front of the screen at the middle of each target location. The animals were trained to reach with the left paw to the left side of the divider and with the right paw to the right side of the divider. A capacitive sensor was placed to either side of each target location, such that the left and right paws activated unique sensors even when both paws reached to the same target. Animals were monitored in the testing room at all times using an infrared camera equipped with an infrared illuminator.

Behavioural procedure. The animals performed delayed saccades or reaches with the left, right, or both arms [Mooshagian, E., Wang, C., Ferdoash, A., Snyder, L.H., Movement order and saccade direction affect a common measure of eye-hand coordination in bimanual reaching, *J. Neurophys.* **112**, 730 (2014)]. Animals first fixated on a circular white stimulus (1.5x1.5°) centred on the screen in front of them. Left and right paws touched "home" pads situated at waist height and 20 cm in front of each shoulder. After 500 ms of holding the initial eye (+/-3°) and hand positions, either one or two peripheral target(s) (5x5°) appeared on the screen. When two targets appeared, they were at opposite locations relative to the fixation point, e.g., left/right or up/down (see below). After an additional 1,250 to 1,750 ms, the central eye fixation target shrank in size to a single pixel, cueing the animal to move to the peripheral target(s) in accordance with a code conveyed by target colour. A green target instructed a left forelimb reach, a red target instructed a right forelimb reach, a blue target instructed a combined reach with both arms, and a white target instructed a saccade (no reach). Trials could be unimanual or bimanual. Bimanual trials could have a single target ("bimanual together") or two targets separated by 180° ("bimanual apart"). All trial types were interleaved. On saccade and unimanual reach trials, the unused hand(s) were required to remain on the home button(s) throughout the trial. On reach trials, eye movements were unconstrained once the go cue appeared. On bimanual trials, the left and right paws were required to hit their target(s) within 500 ms of one another. Spatial tolerances were +/-3° for reaches and +/-2° for saccades. When an error occurred (a failure to achieve or maintain fixation or to touch the home buttons throughout the delay period or a movement that did not achieve the required spatial tolerance), the trial was aborted, and a short (1,500 ms) timeout ensued. Aborted trials were excluded from further analyses. Successful trials were rewarded with a drop of water or juice. Data were collected during 67 and 53 sessions in Monkey 1 and Monkey 2, respectively.

Electrophysiological procedure. Recordings were made from both hemispheres of 2 adult male rhesus monkeys. Recording chambers were centred at approximately 11mm posterior to the ear canals and 8mm lateral of the midline and placed flush to the skull. Extracellular recordings were made using glass-coated tungsten electrodes (Alpha Omega; electrode impedance 0.5–3.0 MΩ). Neurophysiological signals were filtered, sorted, and stored using the Plexon (Dallas, TX) system. Neurons were recorded along the caudal portion of the intraparietal sulcus (IPS). PRR does not fit neatly into any single anatomical area, but instead lies at the boundary of MIP and PO/V6A, though it

also extends slightly towards the lateral bank, towards lateral occipital-parietal (LOP) area (Ref. [37] in the main manuscript and in [Mooshagian, E., Wang, C., Holmes, C. D., and Snyder, L. H. Single units in the posterior parietal cortex encode patterns of bimanual coordination. *Cereb. Cortex* **28**, 1549–1567 (2018)]. We therefore functionally define PRR as that region of cortex containing a large proportion of neurons with visual transients and with sustained delay activity that is substantially greater for combined reaches plus saccades compared with saccades alone in most cases. This functional definition covers much of anatomical areas PO and V6a, on the medial bank of the IPS and rostral bank of the parieto-occipital sulcus, the posterior half of the medial intraparietal area (MIP) on the medial bank, and a portion of LOP on the lateral bank. This definition distinguishes PRR from nearby lateral intraparietal area (LIP), which lies on the lateral bank immediately rostral to LOP and where most neurons show similar responses to combined reaches plus saccades compared with saccades alone.

While searching for neurons, animals performed saccade and right arm only (contralateral) trials as described above. Previous work established that few neurons are active for ipsilateral but not contralateral reaches, and even those neurons are somewhat active during saccades [Chang, S.W., Dickinson, A.R., Snyder, L.H., Limb-specific representation for reaching in the posterior parietal cortex. *J. Neurosci.* **28**, 6128 (2008)]. Online, the preferred direction was defined as the target location that resulted in the largest sustained firing during the delay period for the single target reach conditions (contralateral arm, ipsilateral arm, both arms together). The null direction was defined as the target location 180° from the preferred direction relative to the central fixation point. The preferred direction of the neurons with data for all directions was confirmed by offline analysis. We computed the modulation for each condition for each neuron as the activity for a movement in the preferred direction minus activity for a movement in the null direction. A single preferred direction was determined for each neuron and applied to all tasks.
